# Traumatic brain injury to primary visual cortex produces long-lasting circuit dysfunction

**DOI:** 10.1038/s42003-021-02808-5

**Published:** 2021-11-17

**Authors:** Jan C. Frankowski, Andrzej T. Foik, Alexa Tierno, Jiana R. Machhor, David C. Lyon, Robert F. Hunt

**Affiliations:** 1 Department of Anatomy & Neurobiology, University of California, Irvine, CA 92697 USA; 2 Ophthalmic Biology Group, International Centre for Translational Eye Research, Institute of Physical Chemistry, Polish Academy of Sciences, Warsaw, Poland

**Keywords:** Neurodegeneration, Cellular neuroscience, Neurophysiology

## Abstract

Primary sensory areas of the mammalian neocortex have a remarkable degree of plasticity, allowing neural circuits to adapt to dynamic environments. However, little is known about the effects of traumatic brain injury on visual circuit function. Here we used anatomy and in vivo electrophysiological recordings in adult mice to quantify neuron responses to visual stimuli two weeks and three months after mild controlled cortical impact injury to primary visual cortex (V1). We found that, although V1 remained largely intact in brain-injured mice, there was ~35% reduction in the number of neurons that affected inhibitory cells more broadly than excitatory neurons. V1 neurons showed dramatically reduced activity, impaired responses to visual stimuli and weaker size selectivity and orientation tuning in vivo. Our results show a single, mild contusion injury produces profound and long-lasting impairments in the way V1 neurons encode visual input. These findings provide initial insight into cortical circuit dysfunction following central visual system neurotrauma.

## Abstract (web summary)

Jan Frankowski and Andrzej Foik et al. characterized neuroanatomical, electrophysiological, and functional deficits in the primary visual cortex of mice after traumatic brain injury (TBI). Their results suggest that TBI produces long-lasting impairments to V1 neurons, and provide further insight into circuit dysfunction following injury of the visual cortex.

## Introduction

Posterior impact injuries to the occipital cortex are extremely common in human. Traumatic brain injury (TBI) can lead to long-lasting visual impairments, such as visual acuity and field loss, binocular dysfunction, and spatial perceptual deficits^1–3^, and as many as 75% of military Service members live with permanent visual dysfunction or cortical blindness resulting from a TBI^3^. Restrictive lesions applied to the visual cortex have been shown to trigger cortical plasticity and functional disturbances^4–6^. However, TBI involves mechanical brain damage and a wide range of cortical network abnormalities including cell death, inflammation, and synaptic circuit remodeling^7^. There is essentially nothing known about how visual circuit function is affected by TBI.

Following TBI in human, histological studies have documented a reduction in the number of neurons in the hippocampus^8^ and neocortex^9^. In nonhuman animal models, TBI produces region- and subtype-specific reductions of neurons in various brain areas^10–21^, dramatic circuit rewiring (^22–31^), and a loss of inhibition that does not recover with time^20,21,27,32–39^. However, nearly all of the information about neocortical responses to TBI comes from studies evaluating somatosensory, motor, or frontal cortex. Each of these areas receives numerous intra- and inter-hemispheric inputs from throughout the topographic map^40–42^, whereas callosal connectivity of the visual cortex is limited to the vertical meridian representation along the V1 border^43,44^. Therefore, a deeper understanding of functional disturbances in the brain-injured visual cortex is important, because it has the potential to provide a rational basis for the development of circuit-level therapies for visual cortex injury.

To produce central visual system TBI in adult mice, we applied a focal controlled cortical impact (CCI) injury to the primary visual cortex (V1). We show that although mild contusion injury did not produce a sizable lesion, there was a subtype- and layer-specific loss of neurons in the brain-injured V1. Then, using in vivo electrophysiological recordings of visually evoked responses, we found that mild contusion injury chronically impairs the response of V1 neurons to a variety of visual stimuli. These findings suggest there are profound long-lasting impairments in visual circuit function that result from a single, mild contusive injury to the central visual system. As an initial characterization of central visual system neurotrauma, our results also lay the foundation for future mechanistic investigations of altered cortical network activity and preclinical studies to restore circuit function in the traumatically injured visual cortex.

## Results

### Occipital CCI produces a mild contusion in V1

To evaluate the effect of a single, mild contusion injury to the central visual system, we delivered mild CCI injury centered over the rostral end of V1 in young-adult mice at P60 (Supplementary Fig. 1). We selected CCI as a model, because the injury is highly reproducible from animal to animal, reliably recapitulates structural and functional deficits of TBI and focal contusion injuries are among the most common posterior impact injuries observed in human^1–3^. In all CCI-injured animals (*N* = 7 mice), the lesion consisted of mild tissue compression that was restricted to superficial layers of the cortex at the injury epicenter (Supplementary Fig. 2).

**Fig. 1:**
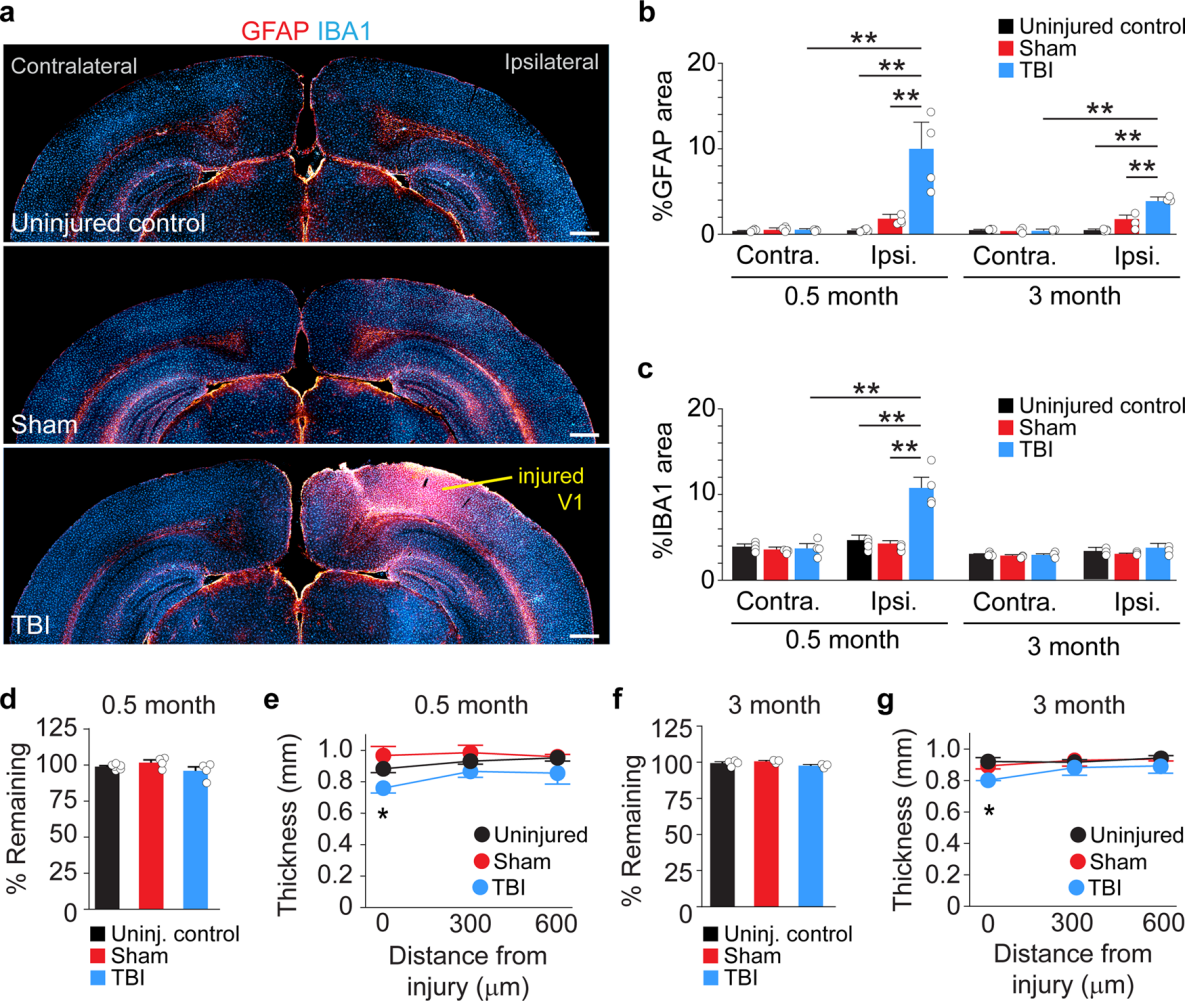
Visual cortex TBI produces a mild cortical lesion. **a** Coronal sections of GFAP (red) and IBA1 (blue) labeling in a control animal and 0.5 months after sham or CCI injury. **b** Quantification of GFAP expression in V1 at 0.5 and 3 months postinjury. **P = 2.9E-07, ipsilateral control versus ipsilateral TBI; **P = 1.3E-05, ipsilateral sham versus ipsilateral TBI; **P = 1.3E-06, ipsilateral TBI versus contralateral TBI at 0.5 months; **P = 1.3E-06, ipsilateral control versus ipsilateral TBI; **P = 8.7E-05, ipsilateral sham versus ipsilateral TBI; **P = 6.0E-06, ipsilateral TBI versus contralateral TBI at 3 months two-way ANOVA with Tukey’s post hoc test, *N* = 3–6 mice per group. **c** Quantification of IBA1 expression in V1 at 0.5 and 3 months postinjury. **P = 2.4E-08, ipsilateral control versus ipsilateral TBI, **P = 2.9E-08, ipsilateral sham versus ipsilateral TBI, **P = 2.3E-08, ipsilateral TBI versus contralateral TBI at 0.5 months; two-way ANOVA with Tukey’s post hoc test, *N* = 3–6 mice per group. **d** Quantification of cortical tissue volume in control, sham, and CCI-injured mice 0.5 months post-CCI. **e** Average thickness of cortex with distance from the injury 0.5 months post-CCI. *P = 0.048, Control versus TBI, two-way repeated-measures ANOVA with Tukey’s post hoc test, *N* = 4–6 mice per group. **f** Quantification of cortical tissue volume in control, sham, and CCI-injured mice 3 months post-CCI. **g** Average cortex thickness with distance from the injury 3 months post-CCI. *P = 0.023, Uninjured versus TBI, two-way repeated-measures ANOVA with Tukey’s post hoc test, *N* = 3–4 mice per group. Scale bars, 500 µm; error bars, SEM.

To define the lesion location, we examined glial responses in V1 following mild CCI injury (Fig. 1a). To do this, we performed an immunostaining analysis at 0.5 months and 3 months after injury for glial fibrillary acidic protein (GFAP), a marker of astrocytes, and ionizing calcium-binding adaptor molecule 1 (IBA1), a marker of activated microglia. In brain-injured animals, the impact site could be clearly identified by a dense pattern of GFAP and IBA1 staining in V1 ipsilateral to the injury. A significant increase in GFAP expression was found in V1 surrounding the injury at 0.5 months, as compared to uninjured controls, sham animals that received a craniotomy but no injury and contralateral tissue sections (Supplementary Fig. 3), and it remained significantly elevated 3 months post-CCI (Fig. 1b). IBA1 immunostaining was also significantly increased ipsilateral to the injury, but only at 0.5 months after injury (Fig. 1c). Uninjured and sham controls did not have an identifiable cortical lesion in any animal.

At 0.5 months post-CCI, a time point when lesion volume is considered to be largely stable^15,45^, there was no significant difference in cortical volume between uninjured control and brain-injured littermates (TBI: 96 ± 3%, sham: 102 ± 2%, compared to 99 ± 1% in uninjured control; *P* = 0.15; one-way ANOVA; *N* = 4–6 mice per group; Fig. 1d). However, when we evaluated the thickness of cortical tissue remaining in the contused portion of the visual cortex, we found a 14% decrease in cortical thickness in brain-injured animals at the injury epicenter, as compared to controls (uninjured: 883 ± 25 μm, sham: 966 ± 58 μm, TBI: 760 ± 32 μm, *P* = 0.048; two-way rmANOVA; *N* = 3–4 mice per group; Fig. 1e). This difference was only observed at the injury epicenter (0 μm); no difference in cortical thickness was observed in tissue sections 300 and 600 μm caudal to the epicenter. We found a similar degree of mild tissue loss at 90d post-CCI (Fig. 1f, g). Thus, CCI produced a mild focal injury with minimal structural damage to V1.

### Neuron loss after V1 injury

**Fig. 2:**
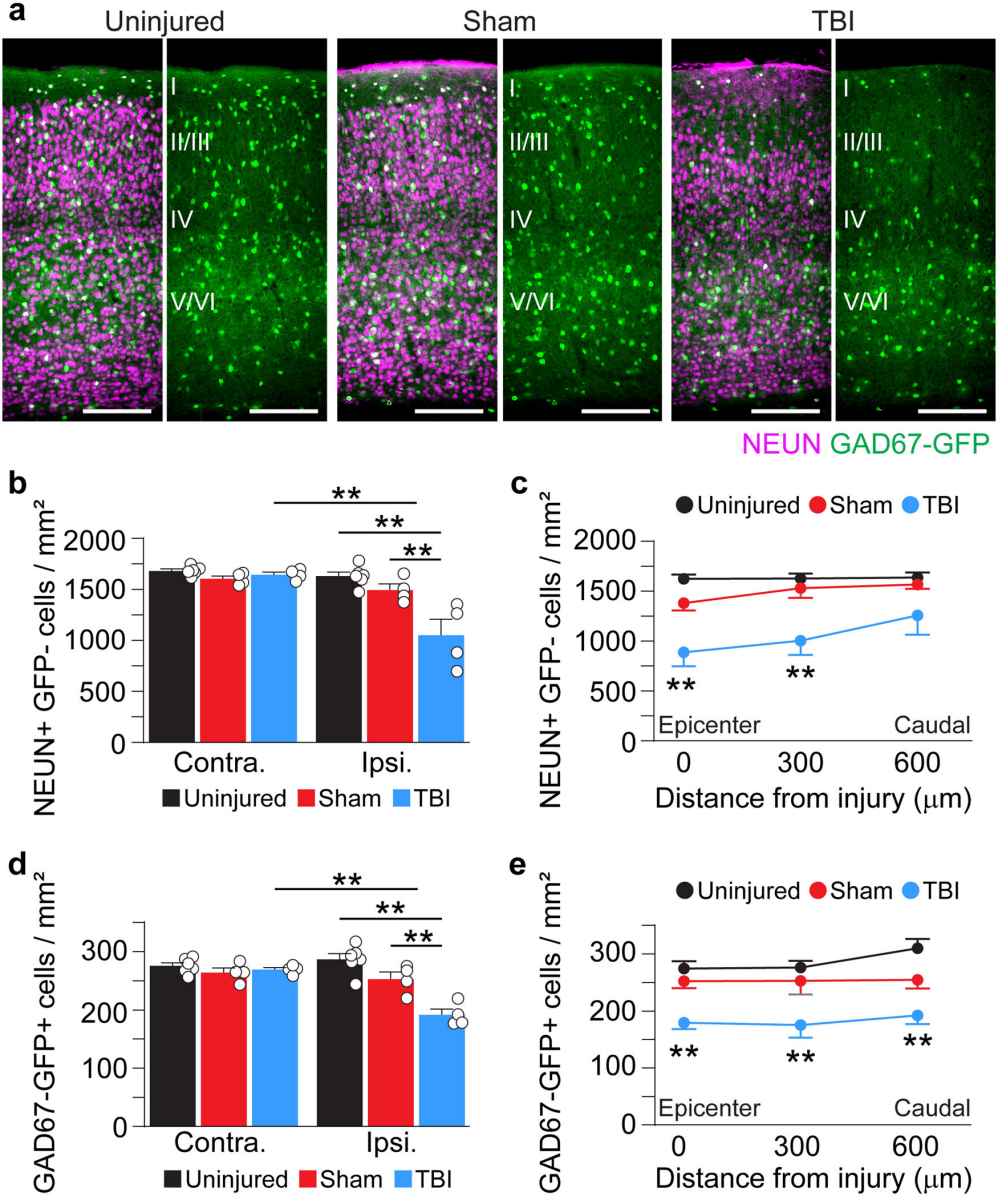
Neuron loss in V1 0.5 months after TBI. **a** Coronal images of control, sham, and CCI-injured V1 labeled for NEUN (magenta) and GAD67-GFP (green). **b** Quantification of NEUN+/GFP- cell density in uninjured control, sham, and brain-injured mice 0.5 months after CCI. **P = 1.79E-05, ipsilateral control versus ipsilateral TBI, **P = 1.77E-03, ipsilateral sham versus ipsilateral TBI, **P = 4.56E-05, ipsilateral TBI versus contralateral TBI; two-way ANOVA with Tukey’s post hoc test, *N* = 4–6 mice per group. **c** NEUN+/GFP- cell density at 0–600 µm from the injury epicenter. **P = 7.86E-05, control versus TBI (0 μm), **P = 1.01E-03, control versus TBI (300 μm); two-way repeated-measures ANOVA with Tukey’s post hoc test; N = 4–6 mice per group. **d** Quantification of GAD67-GFP+ cell density in uninjured control, sham, and brain-injured mice 0.5 months after CCI. **P = 1.07E-06, ipsilateral control versus ipsilateral TBI, **P = 1.58E-03, ipsilateral sham versus ipsilateral TBI, **P = 9.25E-05, ipsilateral TBI versus contralateral TBI, two-way ANOVA with Tukey’s post hoc test, *n* = 4–6 per group. **e** GAD67-GFP+ cell density at 0–600 µm from the injury epicenter. **P = 3.53E-03, control versus TBI (0 μm), **P = 1.66E-03, control versus TBI (300 μm), **P = 1.74E-04, control versus TBI (600 μm); two-way repeated-measures ANOVA with Tukey’s post hoc test; *N* = 4–6 mice per group. Scale bars, 500 μm; error bars, SEM. See Supplementary Data 1 for statistical analyses.

Next, we quantified neuron density in V1 using GAD67-GFP reporter mice that label nearly all GABAergic neurons^46^. Sections were immunostained for GFP to identify inhibitory interneurons and NEUN to identify putative excitatory neurons (i.e., NEUN-positive/GAD67-GFP-negative) (Fig. 2). At 0.5 months after TBI, we found a ~35% reduction in NEUN + /GAD67-GFP- cell density in V1 ipsilateral to the injury (Fig. 2a, b**;** Supplementary Data 1). The reduction in excitatory neurons was most profound at the injury epicenter (45% reduction after TBI) and rapidly decreased with distance away from the impact site (Fig. 2c). We also observed ~35% decrease in the overall density of GAD67-GFP+cells in V1 ipsilateral to the injury (*P* = 1.07E-06, TBI versus uninjured control, two-way ANOVA; Fig. 2d**;** Supplementary Data 1). However, unlike excitatory neurons, GFP + interneuron density was reduced by ~35% at each distance from the impact site (Fig. 2e). No change in cell density was observed in the contralateral hemisphere. These findings suggest mild contusion to the visual cortex produces substantial neuron loss in V1, and the loss of inhibitory neurons is more widespread than excitatory neurons.

**Fig. 3:**
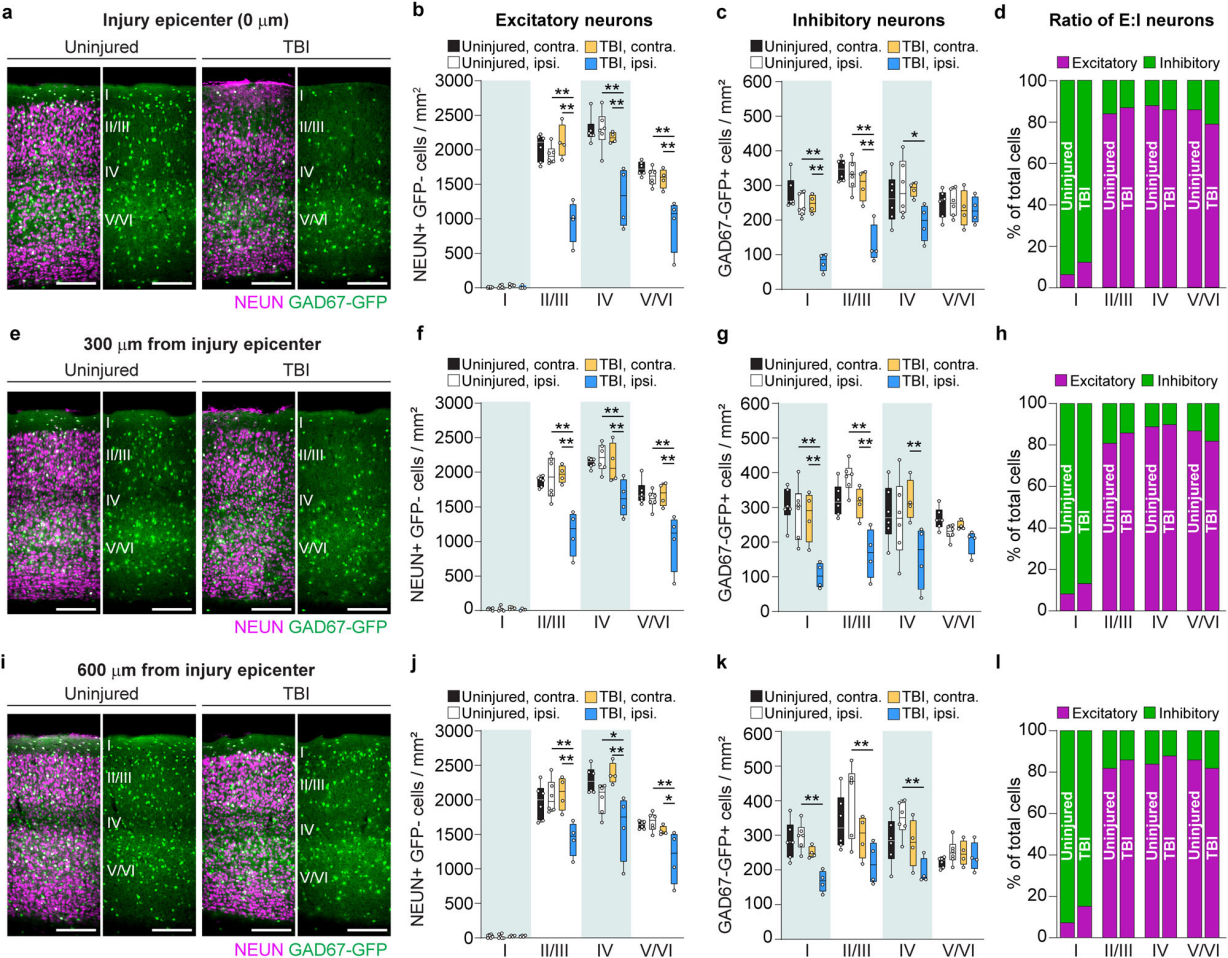
V1 injury produces subtype- and layer-specific loss of neurons. **a, e, i** Coronal images of control and CCI-injured V1 labeled for NEUN (magenta) and GAD67-GFP (green) at 0 (**a**), 300 (**e**), and 600 μm (**i**) from the injury. **b, f, j** Quantification of NEUN+/GFP- cell density in layers I, II/III, IV, and V/VI. *N* = 4–6 mice per group. **c, g, k** Quantification of GAD67-GFP cell density in layers I, II/III, IV, and V/VI. *N* = 4–6 mice per group. **d, h, l** Analysis of the proportion of excitatory to inhibitory neuron density at the injury site (Chi-square = 2.17, df = 3, P = 0.54; **d**), 300 μm (Chi-square = 1.32, df = 3, *P* = 0.72; **h**), or 600 μm caudal to the epicenter (Chi-square = 2.68, df = 3, *P* = 0.44; **l**). Scale bars, 500 μm. Box and whisker plots show median, 25th and 75th percentiles, and the whisker bars represent maximum and minimum values. **P < 0.01, *P < 0.05; random intercept mixed model with Tukey-Kramer post hoc test. See Supplementary Data 2 for statistical analyses.

To determine if post-traumatic neuron loss was layer-specific, we quantified neuron density in cortical layers I, II/III, IV, and V/VI of brain-injured and uninjured control littermates (Fig. 3**;** Supplementary Data 2). For this analysis, we fitted a random intercept mixed model for each cell type to account for the distance from the injury, layer, and treatment condition. We found that excitatory cell loss extended throughout the cortical column ipsilateral to the injury, with significant reductions in NEUN+/ GAD67-GFP- cells in cortical layers II/III, IV, and V/VI (Fig. 3b, f, j); no significant differences were found in layer I where excitatory neurons are rarely found. In contrast, GFP + inhibitory neuron density was most profoundly affected in superficial layers, with significant reductions in GAD67-GFP + neurons in layers I-IV (Fig. 3c, g, k). However, no change in inhibitory neuron density was observed in layers V/VI. Despite these cell-type specific changes in cell density, the ratio of excitatory to inhibitory neurons did not change in any layer of V1 (Fig. 3d, h, l). We conclude that there are subtype- and layer-specific differences in the degree and extent of neuron loss after visual cortex injury.

**Fig. 4:**
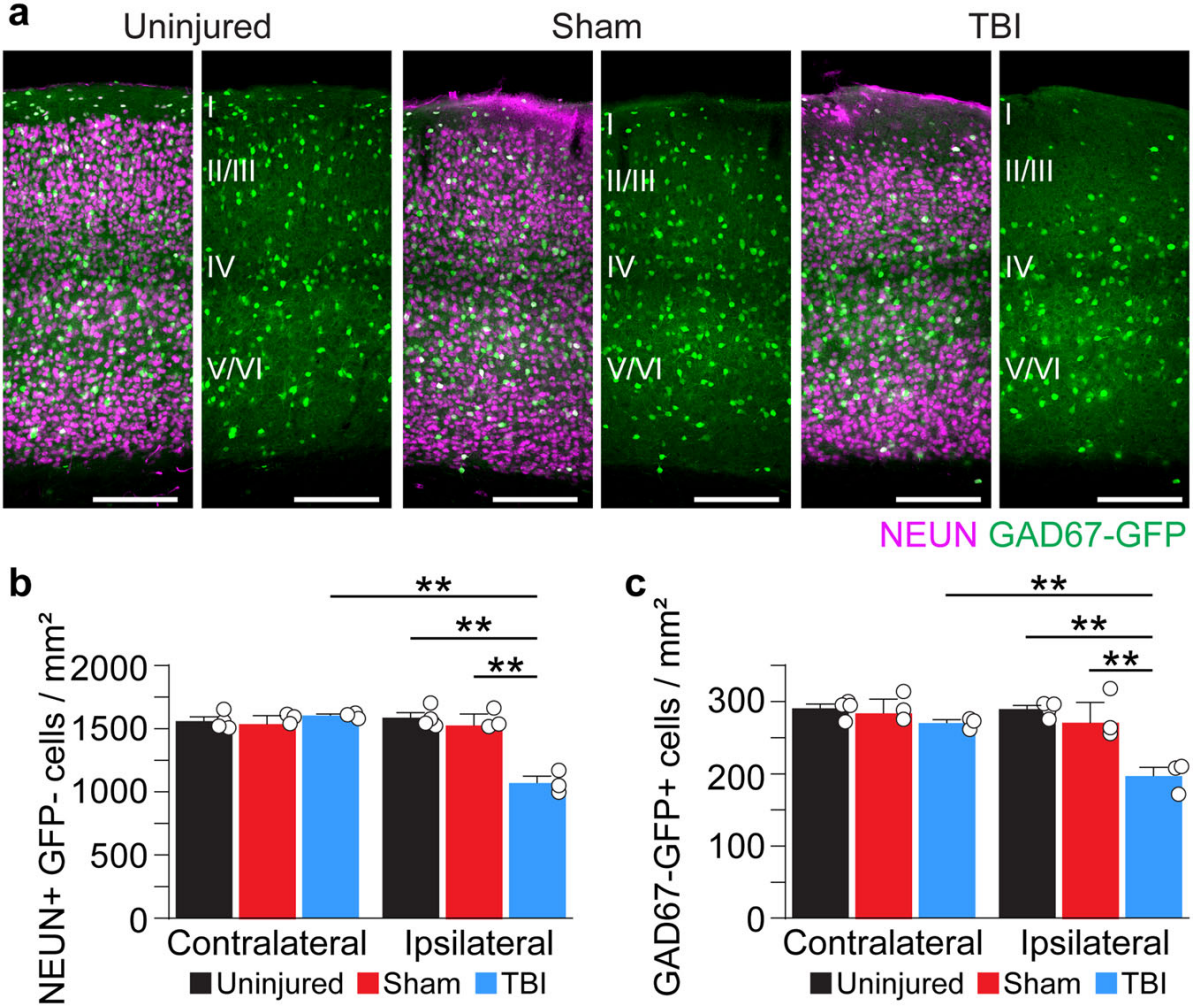
Chronic neuron loss in V1 after TBI. **a** Coronal images of control, sham and CCI-injured V1 labeled for NEUN (magenta) and GAD67-GFP (green) 3 months after TBI. **b** Quantification of NEUN+/GFP- cell density in control, sham, and brain-injured mice 3 months after CCI. **P = 1.33E-06, ipsilateral control versus ipsilateral TBI, **P = 4.23E-06, ipsilateral sham versus ipsilateral TBI, **P = 1.99E-06, ipsilateral TBI versus contralateral TBI; two-way ANOVA with Tukey’s post hoc test, *N* = 3–4 mice per group. **c** Quantification of GAD67-GFP+ cell density in control, sham, and brain-injured mice 90 d after CCI. **P = 1.60E-04, ipsilateral control versus ipsilateral TBI, **P = 1.02E-03, ipsilateral sham versus ipsilateral TBI, **P = 2.96E-03, ipsilateral TBI versus contralateral TBI; two-way ANOVA with Tukey’s post hoc test, *N* = 3–4 mice per group. Scale bar, 500 μm; error bars, SEM. See Supplementary Data 3 for statistical analyses.

We next asked whether the loss of neurons at the injury site persisted long term. At 3 months after injury, NEUN+/GAD67-GFP- cell density remained reduced by 32% ipsilateral to the injury (Fig. 4a, b**;** Supplementary Data 3). GAD67-GFP+ interneuron density was also reduced 3 months post-CCI by 32% (Fig. 4a, c; Supplementary Data 3). These data reproduce our observations at 0.5 months and are consistent with a chronic loss of excitatory and inhibitory neurons after mild contusion injury to the visual cortex.

### Early and long-term disruption of visually evoked responses after TBI

**Fig. 5:**
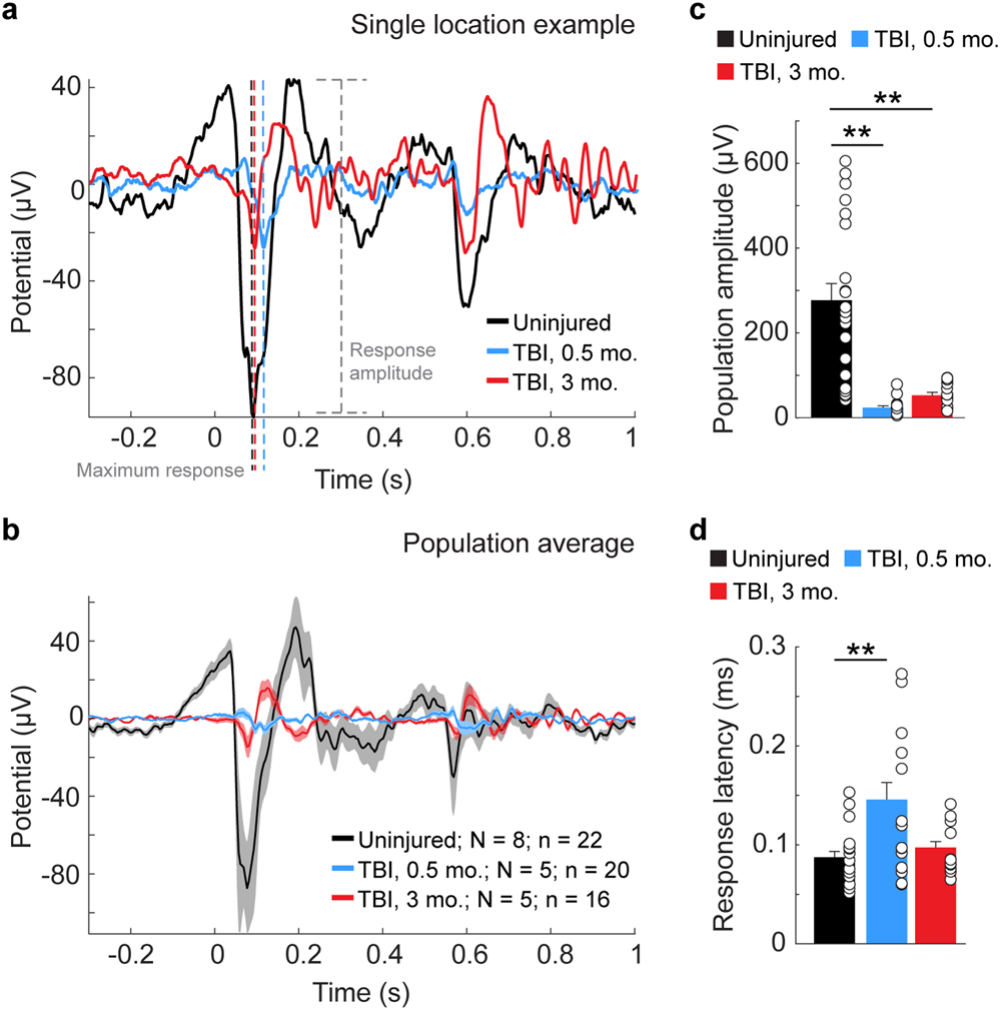
TBI disrupts V1 responses to visual stimuli. **a** Representative example of VEPs in layer 5 of an uninjured control animal (black trace) and animals 0.5 months (blue trace) and 3 months after CCI (red trace). The maximum response for each trace is indicated by dotted black, blue and red lines. Response amplitude for the control condition is indicated by the dotted gray line. **b** Average evoked potentials from recording sites in uninjured control (black) and 0.5 months (blue) and 3 months (red) after TBI. *N* = number of animals; *n* = number of recording locations. Shading indicates S.E.M. **c** Quantification of average evoked amplitude. **P = 6.23E-09, control versus 0.5 months after TBI; **P = 1.88E-03, control versus 3 months after TBI; Kruskal–Wallis H with Dunn’s post hoc. **d** Quantification of average response latency. **P = 0.02, control versus 0.5 months after TBI, Kruskal–Wallis H with Dunn’s post hoc. Individual data points represent the value for each of the recording locations. N, animals; n, recording location; error bars, SEM.

**Fig. 6:**
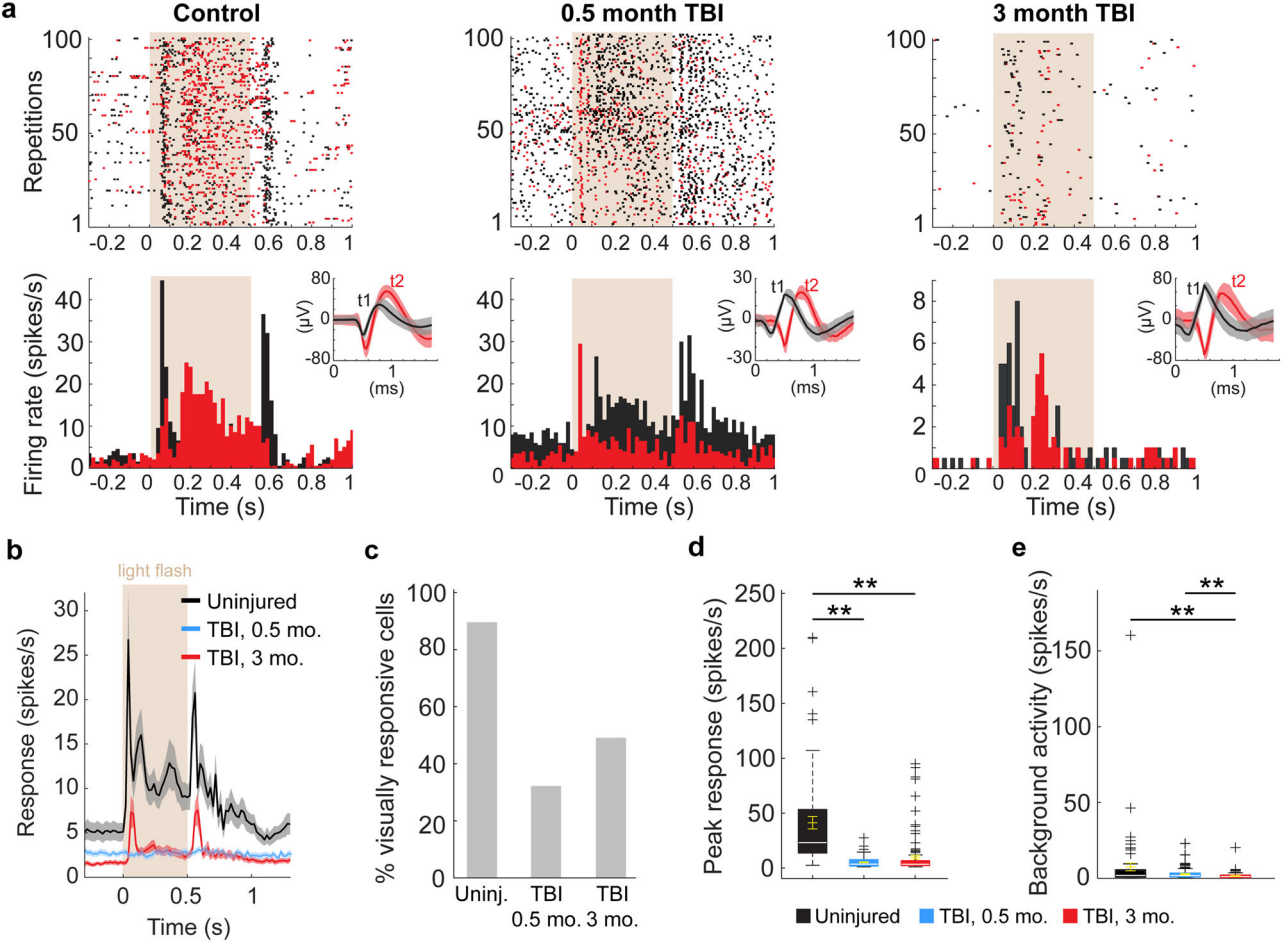
Reduced V1 neuron firing following TBI. **a** Light-evoked responses of action potential firing for two example neurons (black and red) for each animal group: control (left), 0.5 months (middle) and 3 months after injury (right). The top row shows raster plots to 100 repetitions of the flash stimulus. The bottom row shows the spikes/s averaged over 20 ms bins. In both rows, the 500 ms light stimulus is indicated by beige background shading. Insets in the upper right of the bottom row show raw wave forms isolated by two templates (t1, black and t2, red) based on differences in spike amplitude (uV) and timing (ms). Shading indicates spike variability. **b** Population averages of light-evoked single-unit responses of action potential firing in uninjured controls (black) and CCI-injured mice 0.5 months (blue) and 3 months after injury (red). *n* = 67 cells from 8 controls, 110 cells from 5 mice 0.5 months after TBI, and 115 cells from 5 mice 3 months after TBI. Shading indicates S.E.M. The 500 ms light stimulus is indicated by beige background shading. **c**. Percentage of visually responsive cells. **d** Quantification of peak single-neuron firing rates from each group in response to light stimulus. **P = 9.56E-10, control versus 0.5 months after TBI, **P = 9.56E-10, control versus 3 months after TBI; Kruskal–Wallis H with Dunn’s post hoc. **e** Quantification of single-neuron firing rates from each group in the 500 ms prior to the light stimulus. **P = 2.00E-03, control versus 0.5 months after TBI, **P = 3.02E-04, control versus 3 months after TBI; Kruskal–Wallis H with Dunn’s post hoc. For box plots, dashed error bars represent the maximum and minimum observations within 1.5 inter-quartile range of the 25th and 75th percentile; values greater than 1.5 inter-quartile range of the 75th percentile are indicated by +.

**Fig. 7:**
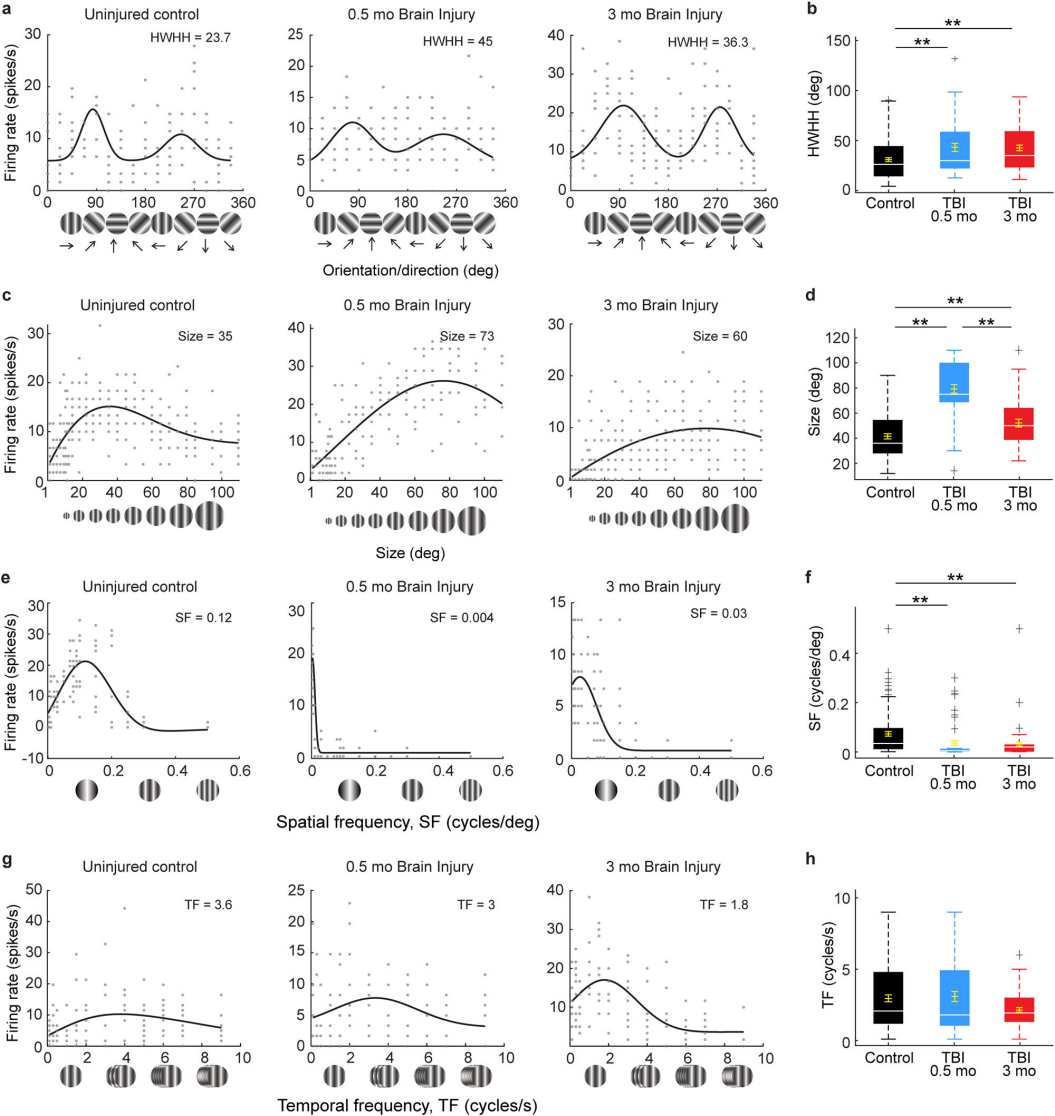
TBI disrupts V1 neuron tuning curves in response to drifting gratings. **a, b** Orientation tuning curves for single neurons in an uninjured control (black) and CCI-injured mice 0.5 months (blue) and 3 months (red) after injury. To facilitate comparisons across examples orientation preferences have been aligned to 90° and 270°, with 90° representing the preferred direction. Tuning values are given as the half-width at half-height (HWHH) in degrees in each panel (**a**) and the population averages are quantified in (**b**). **P = 0.03, control versus 0.5 months after TBI, **P = 1.99E-03, control versus 3 months after TBI; Kruskal–Wallis H with Dunn’s post hoc. *n* = 109 cells from 7 uninjured controls, 54 cells from 5 mice 0.5 months after TBI, and 75 cells from 5 mice 3 months after TBI. **c**, **d** Single neuron examples and population average quantification of aperture size (in degrees). **P = 9.56E-10, control versus 0.5 months after TBI, *P = 0.042, control versus 3 months after TBI, **P = 2.04E-05, 0.5 versus 3 months after TBI; Kruskal–Wallis H with Dunn’s post hoc. *n* = 81 cells for control, 45 cells 0.5 months after TBI and 41 cells 3 months after TBI. **e**, **f** Single neuron examples and quantification of spatial frequency (SF). **P = 3.80E-06, control versus 0.5 months after TBI, **P = 6.51E-03, control versus 3 months after TBI, Kruskal–Wallis H with Dunn’s post hoc. *n* =105 cells for control, 55 cells 0.5 months after TBI, 71 cells 3 months after TBI. **g**, **h** Single neuron examples and quantification of temporal frequency (TF). *P* = 0.26; Kruskal–Wallis H test. Optimal values for each parameter are given in each panel. *n* = 95 cells for control, 59 cells 0.5 months after TBI, and 70 cells 3 months after TBI. Background activity for each cell is indicated by gray dashed lines. For box plots, dashed error bars represent the maximum and minimum observations within 1.5 inter-quartile range of the 25th and 75th percentile; values greater than 1.5 inter-quartile range of the 75th percentile are indicated by +.

To evaluate the in vivo functional state of the visual cortex following TBI, we measured visually evoked potentials (VEPs) and single-unit responses to a range of stimuli across a wide extent of injured V1 at 0.5 and 3 months after injury (Figs. 5–7**)**. First, we recorded VEPs in response to brief flashes of light. These local field potential responses represent the electrical response of a population of V1 neurons to light stimuli. Representative examples of flash-evoked responses are shown in individual animals (Fig. 5a), along with group averages (Fig. 5b). Compared to uninjured controls, evoked VEP amplitudes were significantly reduced by more than 80% in brain-injured mice (control: 277 ± 39 µV, 0.5 months after TBI: 24 ± 4 µV, 3 months after TBI: 53 ± 7 µV; *P* = 9.95E-09, Kruskal–Wallis H test; Fig. 5c), and response latencies rose to more than 60% longer (control: 88 ± 6 ms, 0.5 months after TBI: 146 ± 17 ms, 3 months after TBI: 100 ± 6 ms; *P* = 0.02, Kruskal–Wallis H test; Fig. 5d). Of note, response latencies between light flash and maximal response were longer only at 0.5 months after injury and were similar to controls at 3 months. At both time points, we found that wave profiles in the injured brain lacked a negative wave component normally present in deeper cortical layers (Supplementary Fig. 4).

Single-neuron responses to the same flashes of light were also measured (Fig. 6a**;** Supplementary Fig. 5). Average response profiles showed moderate to negligible activity at both 0.5 and 3 months, respectively, compared to the high average spike rate in control mice (Fig. 6b). After TBI, less than half of the isolated neurons were visually responsive (32% at 0.5 months; 49% at 3 months), compared to 90% of control V1 cells (Chi-square = 56.3, df = 2, *P* = 5.94E-13; Fig. 6c). Similarly, average peak firing rates were significantly lower in brain-injured V1 (control: 42.7 ± 5.7 spikes/s, compared to 5.2 ± 0.4 spikes/s 0.5 months after TBI and 9.9 ± 1.7 spikes/s 3 months after TBI; *P* = 3.6E-20, Kruskal–Wallis H test; Fig. 6d). Prior to stimulation, background activity was highest for the uninjured control group (7.6 ± 2.8 spikes/s) and included one outlier with a baseline firing rate over 150 spikes/second; whereas background activity for cells 0.5 months (2.7 ± 2.5 spikes/s) and 3 months (1.6 ± 2.2 spikes/s) after TBI was significantly lower than in uninjured controls (*P* = 3.59E-20, Kruskal–Wallis H test; Fig. 6e). Together, these findings suggest there is damage to the local V1 neuron population that lasts for several months after TBI.

To evaluate the functional profile of injured V1 in more detail, we next measured single-neuron responses to a range of fundamental visual stimuli, including orientation, size, spatial frequency, and temporal frequency in vivo (Fig. 7; Supplementary Fig. 6). For these analyses, only visually responsive cells were included (see criteria in Methods). Brain-injured mice showed weaker tuning and selectivity to all four types of stimulus parameters compared to uninjured controls (Fig. 7) and had a substantial percentage of cells that were nonresponsive to one or more stimulus conditions (Supplementary Fig. 6). For the cell population, these differences were significant for orientation (Fig. 7b), size (Fig. 7d), and spatial frequency (Fig. 7f), but not temporal frequency (Fig. 7h). The difference was quite striking for orientation and size tuning, both of which are strongly mediated through local cortical inhibition^47–49^. For orientation, the tuning width, measured as the half-width at half-height (HWHH) of the preferred direction (90° in the example cells) was nearly twice as sharp in the control example (23.7°) compared to 0.5 months after injury (45.0°), and more than 50% broader 3 months after injury (36.3°; Fig. 7a). These differences were also seen for the population (control: 30.9° ± 1.9°, 0.5 months after TBI: 43.1° ± 4.0°; 3 months after TBI: 42.6° ± 2.8°; *P* = 0.0013, Kruskal–Wallis H test; Fig. 7b). Broader tuning after TBI is consistent with orientation tuning mediated more through intact thalamocortical feed-forward mechanisms and impairments in cortical inhibition^48,49^. Similarly, the larger size preference in TBI compared to control neuron examples (73° and 60° at 0.5 and 3 months post-TBI vs. 35° in uninjured controls; Fig. 7c) and populations (control: 41.5 ± 2.1°, compared to 79.3 ± 3.4° at 0.5 months and 52.1 ± 3.1° at 3 months postinjury; *P* = 1.16E-13, Kruskal–Wallis H test; Fig. 7d) is also consistent with a loss of cortical inhibition^47^. This is because stimulus size is normally kept small through a process of lateral suppression mediated by long-range intrinsic excitatory V1 neurons synapsing onto local inhibitory neurons^47,50^. We note that the 3 months postinjury group had a statistically smaller preferred size than the 0.5-month group (*P* = 2.04E-05, Kruskal–Wallis H test; Fig. 7d). This could be a sign of recovery, however, a larger percentage of 3-month animal cells did not even respond to the size stimuli (Supplementary Fig. 6c).

## Discussion

Patients with TBI can show long-lasting deficits in visual system function, such as visual acuity and field loss, binocular dysfunction, and spatial perceptual deficits^1^. Here, we delivered a mild focal contusion injury directly to V1 to model occipital contusion injuries, which occur almost exclusively after a direct blow to the back of the head^2,51^. Although V1 was relatively well-preserved, compared to traditional approaches that produce substantial tissue damage^13,16,18^, we found neuron loss at the injury site that extended into deep cortical layers. Interestingly, the degree of neuron loss was different in excitatory versus inhibitory systems. Excitatory neurons were lost throughout all layers of brain-injured V1, but the greatest degree of cell loss was contained at the injury site. In contrast, inhibitory neurons were uniformly lost by ~35% across all sections examined, but cell loss was restricted to superficial layers I-IV of V1. These observations are different from TBI to the hippocampus, where hilar interneurons are widely considered to be the most vulnerable to injury despite being the deepest layer from the site of impact^13,17,20^. The cellular mechanism for these cell-type-specific responses to injury is unknown. In vivo recordings revealed a massive reduction in VEP amplitudes, consistent with damage to the local V1 neuron population, and dramatically altered single-neuron tuning to visual stimuli, including changes in orientation and size, which have been shown to be modulated by cortical interneurons^52^. These findings are consistent with human studies showing visual field dysfunction can occur in individuals with no measurable lesion^53^.

Structural and functional damage following V1 injury appear to be permanent. This is different from damage to other sensory areas. For example, in the whisker barrel cortex, previous in vivo electrophysiology studies have shown there is an initial hypoactivity of neuronal responses 24 h after TBI that recovers within 12 weeks after injury, despite persistent structural changes^28,29^. In the current study, we evaluated the effect of V1 TBI on all GABAergic neurons, but specific subtypes may be more or less vulnerable to injury, as has been seen in other brain areas^13^. Further studies evaluating synaptic plasticity and neuronal connectivity in brain-injured V1 will ultimately be required to determine potential candidate mechanisms underlying the permanent disruption of V1 neuron tuning after TBI.

Individuals with TBI can develop visual impairments independent from other injury-induced motor or cognitive deficits^54–57^. Increases in light intensity evoke inhibitory synaptic activity to prevent changes in luminance intensity from disrupting cortical circuit function^58^ and inability to modulate cortical gain has been proposed as a potential mechanism of injury-related photosensitivity^54,57^. Here we show that basic visual processes in V1 are altered to reflect a loss of cortically mediated inhibition. We found significantly broader orientation tuning widths consistent with reduced local inhibitory neuron activity^52^. Instead, in brain-injured animals, V1 orientation tuning resembles the broader widths mediated through feed-forward mechanisms from the thalamus^48,49^, which are likely more intact. Similarly, increased spatial summation indicated by larger stimulus size preference in TBI is consistent with the loss of local inhibitory neurons mediating surround suppression^59^ and likely reflects preservation of feed-forward mediated mechanisms^47^.

In V1, GABAergic inhibition is essential for a wide range of basic V1 functions, such as tuning a neuron’s preference for stimulus contrast, size, and orientation^52,60,61^, as well as higher-order processing, such as contrast perception^62^. During development, cortical inhibition modulates critical periods, a transient time of enhanced sensitivity to sensory experience. This has been most extensively studied in juvenile V1, in which obstructing vision through one eye results in cortical blindness to this eye, even after normal vision is restored^63^. Cortical inhibition is required for opening the developmental critical period in the visual cortex^64^ and inactivating interneurons can prolong the critical period^65^ or impair cortical plasticity^66^. Even in adulthood, after binocular vision is well established, manipulating inhibition through pharmacology^61,67^ or interneuron transplantation^68,69^ can have dramatic effects on cortical plasticity in response to monocular visual deprivation. Given our recent success using interneuron transplantation to treat post-traumatic memory problems and epilepsy^70,71^, future studies evaluating the effect of manipulating excitatory versus inhibitory activity in brain-injured V1 may reveal new avenues for circuit-based therapy.

## Methods

### Animals

Mice were maintained in standard housing conditions on a 12 h light/dark cycle with food and water provided *ad libitum*. All protocols and procedures were approved by and followed the guidelines of the University Laboratory Animal Resources at the University of California, Irvine and adhered to National Institutes of Health Guidelines for the Care and Use of Laboratory Animals. For electrophysiology experiments, we used C57Bl/6 J mice (Jackson Laboratories, cat no. 000664), and for anatomy experiments, we used a hemizygous glutamic acid decarboxylase—enhanced green fluorescence protein (GAD67-GFP) knock-in line^46^ maintained on a CD-1 background for > 10 generations.

### Experimental design

Male and female mice were randomly allocated to experimental groups prior to TBI. Brain injury was performed at P60, and experiments were performed 0.5 or 3 months after TBI. Brain injuries were only considered to be successful if the lesion was found to be centered over the rostral end of V1. Three animals were excluded from the immunostaining analysis, because upon histological inspection the lesion was not found to be centered over the rostral end of V1. No additional animals were generated to replace these mice. All other brain-injured mice survived and remained otherwise healthy until the day of experimentation.

### Controlled cortical impact (CCI)

Unilateral controlled cortical impact was performed as previously described^13,71^, with modifications to the location and depth of injury. Mice were anesthetized with 2% isoflurane until unresponsive to toe-pinch, then placed into a stereotactic frame and maintained on 1% isoflurane. The fur overlying the skull was trimmed and the scalp was scrubbed with betadine before exposing the skull with a midline incision. The skull was rotated 20 degrees counterclockwise along the rostral-caudal axis and the rostral end of the skull was lowered 20 degrees relative to skull-flat. This orientation centered the impactor tip at the rostral end of V1. A ~4-5 mm craniotomy was centered 3 mm lateral to the midline and 3 mm rostral to the lambdoid suture in the right hemisphere. The skull cap was removed leaving the dura intact. A computer-controlled pneumatically driven impactor (TBI-0310, Precision Systems and Instrumentation) with a 3 mm beveled stainless-steel tip was used to deliver a 0.2 mm depth contusive injury perpendicular to the dura at 3.5 m/s velocity and 500 ms of impactor dwell time. The skull cap was not replaced, and the incision was closed with silk sutures. Animals undergoing surgical procedures received buprenorphine hydrochloride (Buprenex, 0.05 mg/kg, delivered i.p.) preoperatively and once daily for 3d. A postoperative health assessment was performed for 5d following surgical procedures.

### Immunostaining

At 0.5 or 3 months after injury, mice were transcardially perfused with 4% paraformaldehyde (PFA) and free-floating vibratome sections (50 µm) were processed using standard immunostaining procedures^71^. Sections were stained with the following primary antibodies: GFP (1:1000; cat. no. GFP-1020, Aves Labs), NEUN (1:1000; cat. no. MAB377, Millipore), GFAP (1:500, cat. no. MAB3402, Millipore) and IBA1 (1:1000, cat. no. 019-19740, Fujifilm). Secondary antibodies were Alexa 488, 546, 594, and 647 (1:1000; cat. nos. A-11039, A-11005, A-11030 and A-21244, Fisher Scientific). Sections were then mounted on charged slides (Superfrost plus; Fisher Scientific) with Fluoromount-G containing DAPI (Southern Biotech). Images were obtained with a Leica DM6 epifluorescence microscope. Brightness and contrast were adjusted manually using Adobe Photoshop; z-stacks were generated using Leica software.

### Volumetric analysis

Quantification of cortical lesion volume was performed by measuring the area of cortical tissue remaining in both hemispheres in eight DAPI-labeled coronal sections along ~2400 μm of the rostral-caudal axis spaced 300 µm apart as previously described^13,71^. Borders of the cortical plate were drawn between the dorsal aspect of the corpus callosum and the pial surface using ImageJ. Regions of the cortical subplate (e.g., amygdala) were excluded from analysis. The % of the ipsilateral cortex remaining for each animal was calculated using the following formula:

$$\% \,{{{{{\rm{Cortex}}}}}}\,{{{{{\rm{Remaining}}}}}}=\left(\,\frac{\,\sum {i}_{n}}{\sum {c}_{n}}\,\right)\times 100$$

where *i* = the area of the ipsilateral cortex and *c* = the area of the contralateral cortex and *n* = the section number.

### Cortical thickness measurement

Average cortical thickness was measured from a series of three DAPI-labeled x10 images of the entire cortical column centered at the injury epicenter and two 300 μm serial sections caudal to the epicenter. The area of tissue between the pial surface and the ventral aspect of layer V/VI was divided by the width of the frame (958.29 µm) to obtain an average cortical thickness value along the width of the frame. For uninjured controls, images were taken in corresponding brain sections at the most central portion of V1 as defined in the 2017 Allen Reference Atlas.

### Cell quantification

Fluorescently labeled coronal brain sections (50 μm) were imaged using a Leica DM6 fluorescence microscope with an x20 objective and quantification was performed in ImageJ, as previously described^13,71^. For quantification of cell density, three brain sections spaced 300 μm apart were counted, with the rostral-most section at the injury epicenter and the next two additional sections caudal to the epicenter. For layer analysis, the border of each layer (layers I, II/III, IV, and V/VI) were defined manually by visual inspection of neuron densities in NEUN epifluorescence images, as previously described^72,73^. For quantification of GFAP and IBA1 immunostaining, measurements were analyzed at three different locations and the percentage of the area above fluorescence threshold was applied using ImageJ according to a previous protocol^71^. The same settings were used for all sections.

### Neurophysiology

Animals were initially anesthetized with 2% isoflurane in a mixture of N_2_O/O_2_ (70%/30%) then placed into a stereotaxic apparatus. A small, custom-made plastic chamber was secured to the exposed skull using dental acrylic. After one day of recovery, re-anesthetized animals were placed in a custom-made hammock, maintained under isoflurane anesthesia (1–2% in N_2_O/O_2_) and multiple single tungsten electrodes were inserted into V1 layers II–VI using the same craniotomy produced during the injury phase. All recording locations were within the CCI damaged region of V1 (defined as being within the craniotomy). Following electrode placement, the chamber was filled with sterile agar and sealed with sterile bone wax. Animals were then sedated with chlorprothixene hydrochloride (1 mg/kg; IM;^74^) and kept under light isoflurane anesthesia (0.2–0.4% in 30% O_2_) throughout the recording procedure. EEG and EKG were monitored throughout and body temperature was maintained with a heating pad (Harvard Apparatus, Holliston, MA).

Data was acquired using a multi-channel Scout recording system (Ripple, UT, USA). Local field potentials (LFP) from multiple locations at matching cortical depths were band-pass filtered from 0.1 Hz to 250 Hz and stored along with spiking data at 1 kHz sampling rate. LFP signal was aligned to stimulus time stamps and averaged across trials for each recording depth in order to calculate visually evoked potentials (VEP)^75–77^. Single-neuron spike signals were band-pass filtered from 500 Hz to 7 kHz and stored at a 30 kHz sampling frequency. Spikes were sorted online in Trellis (Ripple, UT, USA) while performing visual stimulation. Action potentials were detected based on negative and positive thresholds that were at least twice as large (S/N > 2:1) as the background noise. For each recording location, thresholds were adjusted to maintain a high signal-to-noise ratio. Waveforms were sorted by marking templates based on the clear amplitude difference, positive or negative peak detection, and the slope between negative and positive component (see insets in Fig. 6a), which can be defined as the spike width. Visual stimuli were generated in Matlab (Mathworks, USA) using Psychophysics Toolbox^78–80^ and displayed on a gamma-corrected LCD monitor (55 inches, 60 Hz; 1920 ×1080 pixels; 52 cd/m^2^ mean luminance). Stimulus onset times were corrected for monitor delay using an in-house designed photodiode system^81^.

Visual responses were assessed according to previously published methods^76,81,82^. For recordings of visually evoked responses, cells were first tested with 100 repetitions of a 500 ms bright flash stimulus (105 cd/m^2^). Receptive fields for visually responsive cells were then located using square-wave drifting gratings, after which optimal orientation, direction, and spatial and temporal frequencies were determined using sine-wave gratings. Shown at optimal orientation, spatial frequencies used ranged from 0.001 to 0.5 cycles/°; Temporal frequencies used were from 0.1 to 10 cycles/s. Using optimal parameters, size tuning was assessed with apertures ranging from 1 to 110° at 100% contrast. With optimal size, orientation tuning of the cell was re-assessed using 8 orientations × 2 directions each, stepped by 22.5° increments. Background activity was calculated as average activity from 500 ms before stimulus onset for each repetition. A cell was determined to be visually responsive if the average firing rate was more than 2 standard deviations above background activity and at least 3 spikes/s. Any cell that was nonresponsive to the flash stimulus was not probed using sine-wave gratings. A percentage of flash-responsive cells in the 0.5- and 3-month conditions did not respond to every sine-wave stimulus condition used (Supplemental Fig. 6). Non-responses to sine-wave stimuli were excluded from population analyses because they could not be fit to a curve.

### Local field potential (LFP) analysis

Amplitude of response was calculated as a difference between the peak of the positive and negative components of the VEP. Response latency was defined as the time from stimulus onset to maximum response. Maximum of the response was defined at the larger of the negative or positive peak. For uninjured control animals, depths corresponding to layer 5 were always used (~500 μm). This is because layer 5 amplitude responses were the highest in control animals (see example in Supplemental Fig. 3a). For TBI animals, the depth with the highest amplitude was used. This is because VEPs were more erratic in TBI animals and not always the most responsive at layer 5 (see examples in Supplemental Fig. 3b, c).

### Single-unit analysis

Tuning curves were calculated based on the average spike rate centered around the preferred direction (peak response). Optimal visual parameters were chosen as the maximum response value. Orientation tuning was measured in degrees at the half-width at half-height (HWHH; 1.18 × σ) based on fits to Gaussian distributions^47,48,81–84^ using:

1 $${R}_{{O}_{s}}={baseline}+{R}_{p}{e}^{-\frac{{\left({O}_{s}-{O}_{p}\right)}^{2}}{{2\sigma }^{2}}}+{R}_{n}{e}^{-\frac{{\left({O}_{s}-{O}_{p}+180\right)}^{2}}{{2\sigma }^{2}}},$$

where *O*_s_ is the stimulus orientation, *R*_Os_ is the response to different orientations, *O*_p_ is the preferred orientation, *R*_p_ and *R*_n_ are the responses at the preferred and nonpreferred direction, σ is the tuning width, and ‘baseline’ is the offset of the Gaussian distribution. Gaussian fits were estimated without subtracting spontaneous activity, similar to the procedures of Alitto and Usrey^83^.

Size tuning curves were fitted by a difference of Gaussian (DoG) function:

2 $${R}_{s}={K}_{e}{\int }_{-s}^{s}{e}^{{(-\frac{x}{{r}_{e}})}^{2}}{dx}-{K}_{i}{\int }_{-s}^{s}{e}^{{(-x/{r}_{i})}^{2}}{dx}+{R}_{0},$$

in which *R*_s_ is the response evoked by different aperture sizes. The free parameters, *K*_e_ and re, describe the strength and the size of the excitatory space, respectively; Ki and ri represent the strength and the size of the inhibitory space, respectively; and *R*_0_ is the spontaneous activity of the cell.

The optimal spatial and temporal frequency was extracted from the data fitted to Gaussian distributions using the following equation:^81,82,85,86^

3 $${R}_{{SF}/{TF}}={baseline}+{R}_{{pref}}{e}^{-\frac{{\left(\frac{{SF}}{{TF}}-{\frac{{SF}}{{TF}}}_{{pref}}\right)}^{2}}{{2\sigma }^{2}}},$$

Where *R*_SF/TF_ is the estimated response, *R*_pref_ indicates response at preferred spatial or temporal frequency, SF/TF indicates spatial or temporal frequency, σ is the standard deviation of the Gaussian, and baseline is Gaussian offset.

### Statistics and reproducibility

Anatomical data analysis was performed in Graphpad Prism 9, Microsoft Excel, and SAS 9.4 software. Experimental groups were averaged across groups (i.e., *N* = animals) compared by two-way ANOVA with Tukey’s post hoc test, or repeated-measures two-way ANOVA followed by Sidak’s post hoc test. For layer analysis, data were fitted to a random intercept mixed model followed by Tukey-Kramer post hoc. Cell density was defined as the response variable and distance from the injury, cell layer, group, the interaction of layer by group, and the interaction of distance by layer by the group as explanatory variables. Neurophysiology data analysis was performed in Matlab (Mathworks, USA). Neural responses were averaged across recording locations (i.e., *N* = animals; *n* = recording locations) or cells (in single-unit recordings) and groups were compared by Kruskal–Wallis H test followed by multiple comparisons using Dunn’s post hoc. All data are expressed as mean ± SEM. Significance was set at *P* < 0.05.

### Reporting Summary

Further information on research design is available in the Nature Research Reporting Summary linked to this article.

## Supplementary information

Supplementary Information

Description of Additional Supplementary Files

Supplementary Data 1

Supplementary Data 2

Supplementary Data 3

Supplementary Data 4

Reporting Summary

## Data Availability

All data that support the findings of this study are available as source data in Supplementary Data 4. All other data are available from the corresponding author upon reasonable request.

## Code availability

Data were collected with previously published custom MatLab script^76^ and is available from the corresponding author upon reasonable request.
