# Supplementary material for: Traumatic brain injury to primary visual cortex produces long-lasting circuit dysfunction
Source: Commun Biol. 2021 Nov 17;4:1297. doi: 10.1038/s42003-021-02808-5 (PMC8599505; doi:10.1038/s42003-021-02808-5)
Supplement: Supplementary file 1 — Supplementary Information [file 42003_2021_2808_MOESM1_ESM.pdf]

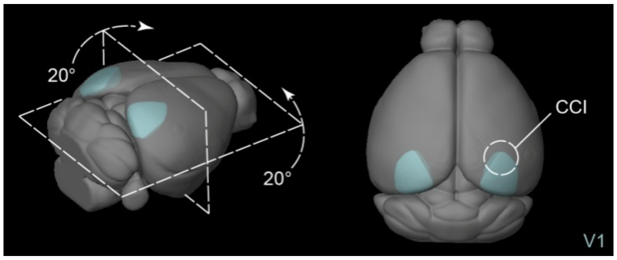

Supplementary Figure 1. Schematic of Allen Mouse Common Coordinate Framework showing the head rotation used to produce CCI injury over V1 (cyan). CCI injury was centered over rostral primary visual cortex (white circle).

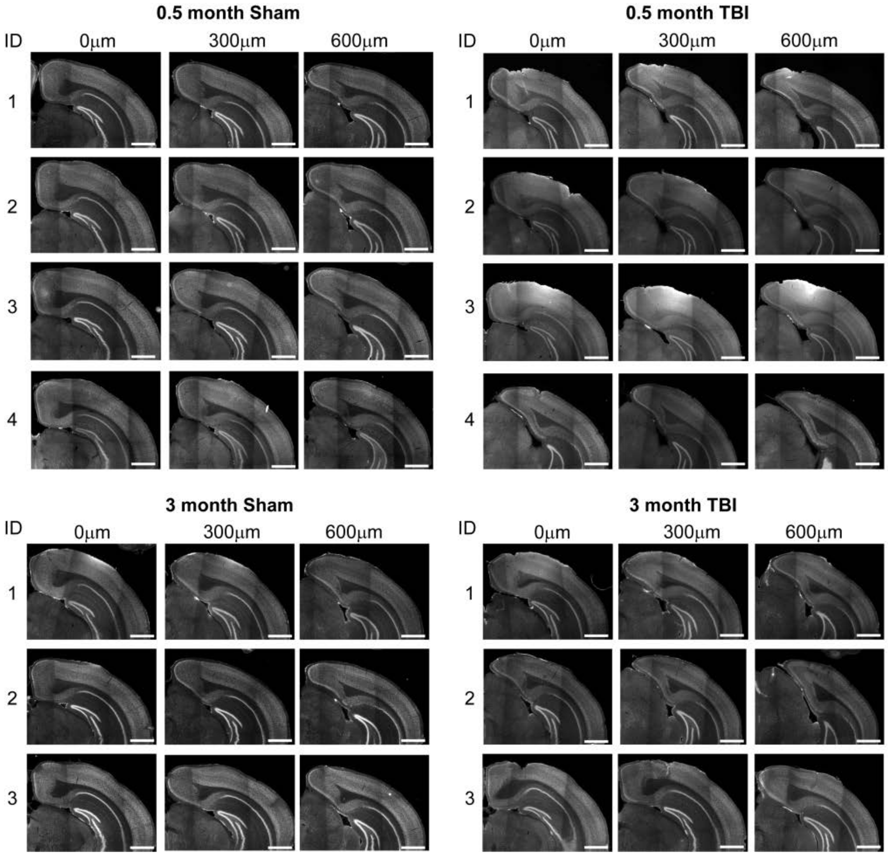

Supplementary Figure 2. Coronal sections of ipsilateral V1 showing NEUN labeling in all sham and brain injured animals used for histological quantifications in this study. ID, animal identification. Scale bars, 1mm.

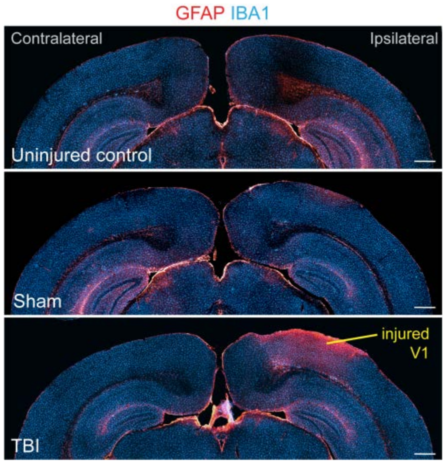

Supplementary Figure 3. Coronal sections of GFAP (red) and IBA1 (blue) labeling in an uninjured control animal and 3 months after sham or CCI injury. Scale bars, 500 µm

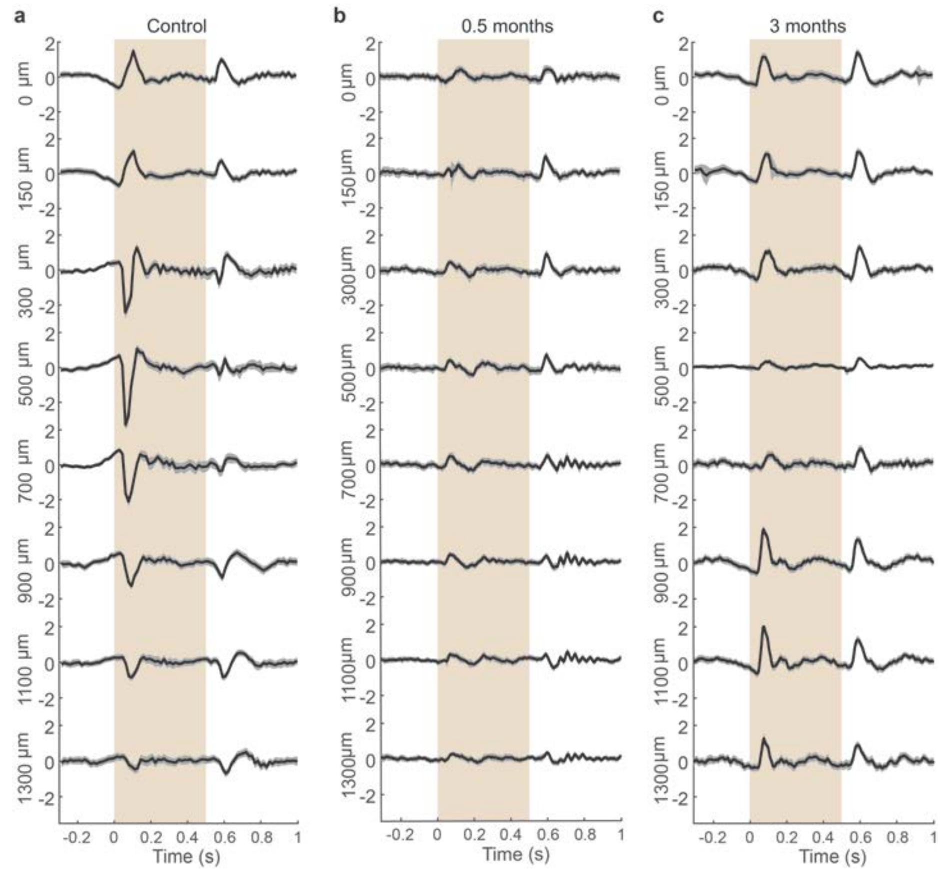

Supplementary Figure 4. a-c. Representative examples of LFP responses through the cortical depth in a control animal (a), and at the site of injury 0.5 months (b) and 3 months (c) after TBI. Grey shading indicates S.E.M. Visual stimulus was presented for 500 ms at Time 0 (beige background shading).

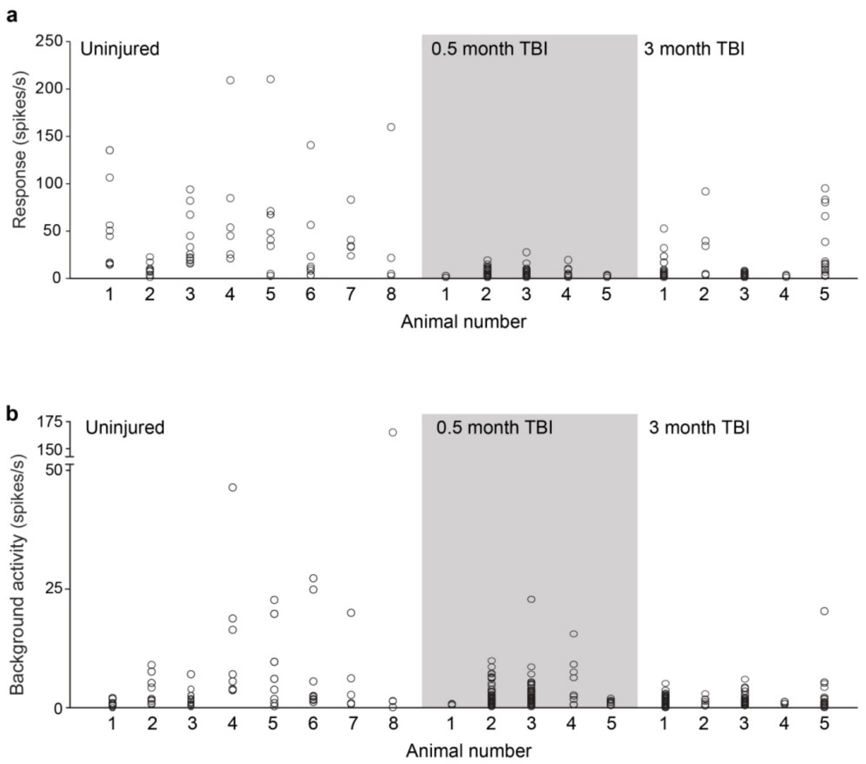

Supplementary Figure 5a,b. Distribution of peak response (a) and background activity (b) for each animal in the uninjured (left, 1-8), 0.5 months (middle, 1-5, gray shading) and 3 months (right, 1-5) after TBI groups.

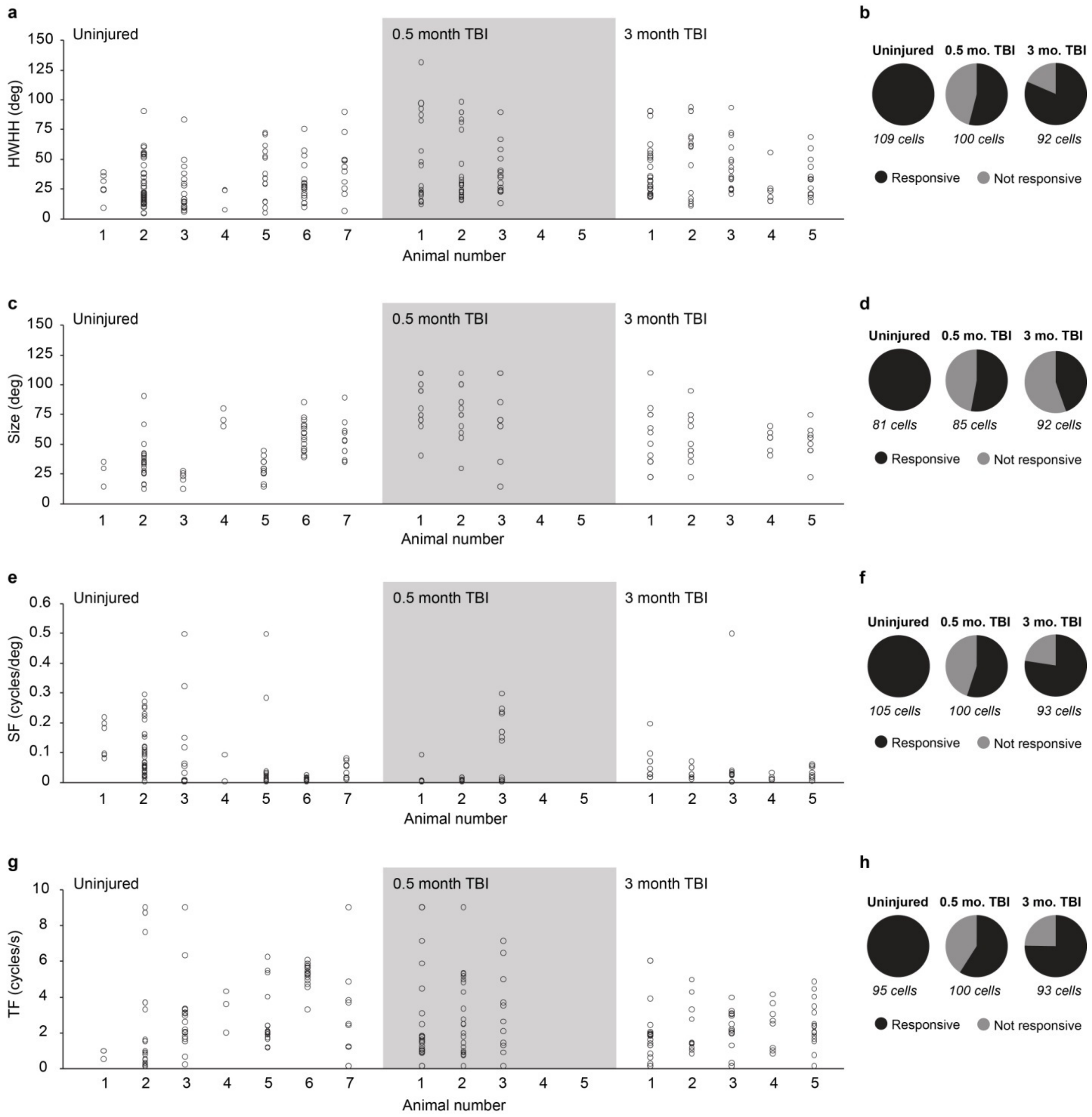

Supplementary Figure 6. a,c,e,g. Distribution of half-width at half-height (a), preferred stimulus size (c), preferred spatial (e) and temporal (g) frequencies for each animal in the uninjured (left, 1-7), 0.5 months (middle, 1-5, gray shading) and 3 months (right, 1-5) after TBI groups. **b,d,f,h.** Proportion of cells that were responsive (black) and not-responsive (gray) to each stimulus parameter: orientation (Chi-square = 67.2, df = 2, P = 3E-15; b), size (Chi-square = 65.7, df = 2,

P = 5E-15; d), spatial frequency (Chi-square = 60.2, df = 2, P = 8.6E-14; f), and temporal frequency (Chi-square = 47.9, df = 2, P = 4E-11; h).
