# Supplementary material for: Traumatic brain injury to primary visual cortex produces long-lasting circuit dysfunction
Source: Commun Biol. 2021 Nov 17;4:1297. doi: 10.1038/s42003-021-02808-5 (PMC8599505; doi:10.1038/s42003-021-02808-5)
Supplement: Supplementary file 2 — Description of Additional Supplementary Files [file 42003_2021_2808_MOESM2_ESM.pdf]

**Description of Additional Supplementary Files**

**File name:** Supplementary Data 1

**Description:** Cell density quantification 0.5 months after TBI.

**File name:** Supplementary Data 2

**Description:** Cell density quantification 3 months after TBI.

**File name:** Supplementary Data 3

**Description:** Laminar density quantification.

**File name:** Supplementary Data 4

**Description:** Source data.
