# Supplementary material for: Traumatic brain injury to primary visual cortex produces long-lasting circuit dysfunction
Source: Commun Biol. 2021 Nov 17;4:1297. doi: 10.1038/s42003-021-02808-5 (PMC8599505; doi:10.1038/s42003-021-02808-5)
Supplement: Supplementary file 7 — Reporting Summary [file 42003_2021_2808_MOESM7_ESM.pdf]

nature research

Corresponding author(s): Robert F Hunt

Last updated by author(s): Oct 4, 2021

Reporting Summary

Nature Research wishes to improve the reproducibility of the work that we publish. This form provides structure for consistency and transparency in reporting. For further information on Nature Research policies, see our Editorial Policies and the Editorial Policy Checklist.

Statistics

For all statistical analyses, confirm that the following items are present in the figure legend, table legend, main text, or Methods section.

n/a	Confirmed	
☐	☒	The exact sample size (*n*) for each experimental group/condition, given as a discrete number and unit of measurement
☒	☐	A statement on whether measurements were taken from distinct samples or whether the same sample was measured repeatedly
☐	☒	The statistical test(s) used AND whether they are one- or two-sided<br>*Only common tests should be described solely by name; describe more complex techniques in the Methods section.*
☐	☒	A description of all covariates tested
☐	☒	A description of any assumptions or corrections, such as tests of normality and adjustment for multiple comparisons
☐	☒	A full description of the statistical parameters including central tendency (e.g. means) or other basic estimates (e.g. regression coefficient) AND variation (e.g. standard deviation) or associated estimates of uncertainty (e.g. confidence intervals)
☐	☒	For null hypothesis testing, the test statistic (e.g. *F*, *t*, *r*) with confidence intervals, effect sizes, degrees of freedom and *P* value noted<br>*Give P values as exact values whenever suitable.*
☒	☐	For Bayesian analysis, information on the choice of priors and Markov chain Monte Carlo settings
☒	☐	For hierarchical and complex designs, identification of the appropriate level for tests and full reporting of outcomes
☐	☒	Estimates of effect sizes (e.g. Cohen's *d*, Pearson's *r*), indicating how they were calculated

Our web collection on statistics for biologists contains articles on many of the points above.

Software and code

Policy information about availability of computer code

Data collection	Leica LAS X, Trellis, Matlab
Data analysis	Graphpad Prism 9, Microsoft Excel, Matlab, SAS 9.4 software

For manuscripts utilizing custom algorithms or software that are central to the research but not yet described in published literature, software must be made available to editors and reviewers. We strongly encourage code deposition in a community repository (e.g. GitHub). See the Nature Research guidelines for submitting code & software for further information.

Data

Policy information about availability of data

All manuscripts must include a data availability statement. This statement should provide the following information, where applicable:

- Accession codes, unique identifiers, or web links for publicly available datasets
- A list of figures that have associated raw data
- A description of any restrictions on data availability

All data that support the findings of this study are available from the corresponding authors upon reasonable request.

Field-specific reporting

Life sciences study design

All studies must disclose on these points even when the disclosure is negative.

Sample size	No statistical methods were used to predetermine sample size. Sample sizes were chosen based on previous literature for similar experiments and effect sizes calculated to examine the magnitude of the experimental effect.
Data exclusions	Three animals were excluded from the immunostaining analysis, because upon histological inspection the lesion was not found to be centered over the rostral end of V1. No additional animals were generated to replace these mice.
Replication	Multiple cohorts of animals were used for each experiment; anatomical and electrophysiology experiments were performed at two distinct time points. All attempts at reproducing our results were successful.
Randomization	Upon arrival, animals were habituated to their housing for up to 1 week before being randomly assigned into uninjured (naive control), sham or TBI treatment groups. Brain injured mice and age-matched controls were housed together (2–5 animals per cage).
Blinding	For anatomical analyses, investigators were blinded to the treatment of the animals. For neurophysiology experiments, investigator blinding was not possible due to the craniotomy present in brain injured animals.

Reporting for specific materials, systems and methods

We require information from authors about some types of materials, experimental systems and methods used in many studies. Here, indicate whether each material, system or method listed is relevant to your study. If you are not sure if a list item applies to your research, read the appropriate section before selecting a response.

Materials & experimental systems

n/a	Involved in the study	
☐	☒	Antibodies
☒	☐	Eukaryotic cell lines
☒	☐	Palaeontology and archaeology
☐	☒	Animals and other organisms
☒	☐	Human research participants
☒	☐	Clinical data
☒	☐	Dual use research of concern

Methods

n/a	Involved in the study	
☒	☐	ChIP-seq
☒	☐	Flow cytometry
☒	☐	MRI-based neuroimaging

Antibodies

Antibodies used	Primary antibodies were GFP (1:1000; cat. no. GFP-1020, Aves Labs), NEUN (1:1000; cat. no. MAB377, Millipore), GFAP (1:500, cat. no. MAB3402, Millipore) and IBA1 (1:1000, cat. no. 019-19740, Fujifilm). Secondary antibodies were Alexa 488, 546, 594 and 647 (1:1000; cat. nos. A-11039, A-11005, A-11030 and A-21244, Fisher Scientific)
Validation	All antibodies have been extensively used for immunostaining analysis in brain.

Animals and other organisms

Policy information about studies involving animals; ARRIVE guidelines recommended for reporting animal research

Laboratory animals	Male and female mice; 2 to 5 months old. For electrophysiology experiments, we used C57Bl/6J mice (Jackson Laboratories, cat no. 000664), and for anatomy experiments, we used a hemizygous glutamic acid decarboxylase - enhanced green fluorescence protein (GAD67-GFP) knock-in line (Tamamaki et al., 2003) maintained on a CD-1 background for > 10 generations.
Wild animals	*Provide details on animals observed in or captured in the field; report species, sex and age where possible. Describe how animals were caught and transported and what happened to captive animals after the study (if killed, explain why and describe method; if released, say where and when) OR state that the study did not involve wild animals.*
Field-collected samples	*For laboratory work with field-collected samples, describe all relevant parameters such as housing, maintenance, temperature, photoperiod and end-of-experiment protocol OR state that the study did not involve samples collected from the field.*
Ethics oversight	*Identify the organization(s) that approved or provided guidance on the study protocol, OR state that no ethical approval or guidance was required and explain why not.*

Note that full information on the approval of the study protocol must also be provided in the manuscript.
